# Interaction between APOE ɛ4 genotype and education on mild cognitive impairment

**DOI:** 10.1093/eurpub/ckac130.082

**Published:** 2022-10-25

**Authors:** M Frank, J Hensel, L Baak, N Dragano, C Weimar, M Jokisch, B Schmidt

**Affiliations:** 1 Institute for Medical Informatics, Biometry and Epidemiology, University of Duisburg-Essen, Essen, Germany; 2 Institute of Medical Sociology, University of Düsseldorf, Düsseldorf, Germany; 3 Center for NeuroRehabilitation, BDH-Clinic Elzach, Elzach, Germany; 4 Department of Neurology, University Hospital Essen, Essen, Germany

## Abstract

**Introduction:**

Studies have identified the apolipoprotein E (APOE) ɛ4 genotype to be a genetic risk factor for mild cognitive impairment (MCI) with environmental factors, such as education, modifying this genetic effect. The aim of this study was to investigate possible interaction of APOE ɛ4 status and educational attainment on MCI.

**Methods:**

Information on education, MCI and APOE status was available for 3,829 participants from the Heinz Nixdorf Recall study. Logistic regression models were fitted to estimate sex- and age-adjusted odds ratios (OR) and 95% confidence intervals (95%-CI) for education (<14 years vs. ≥14 years of education), APOE status (carrier vs. noncarrier) and the interaction between APOExeducation on multiplicative scale with MCI. To consider interaction on additive scale, the relative excess risk due to interaction (RERI) was calculated. The effect of APOE on MCI was additionally stratified by educational groups.

**Results:**

A higher chance of MCI was observed for reporting <14 years of education (OR: 1.37 [95%-CI: 1.11, 1.69]) and having a positive APOE ɛ4 status (OR: 1.27 [95%-CI: 1.04, 1.55]). Stratified analysis showed a stronger genetic effect of APOE ɛ4 status on MCI in participants with low education (OR: 1.42 [95%-CI: 1.12, 1.79)], compared to participants with higher education (OR: 1.00 [95%-CI: 0.67, 1.45)]. An indication for positive interaction between education and APOE ɛ4 status on MCI was found on additive scale (RERI: 0.52 [95%-CI: 0.01, 1.03]), no interaction on the multiplicative scale was observed.

**Conclusions:**

Results gave indication for positive interaction on the additive scale of APOE ɛ4 status and education, showing stronger genetic effects on MCI in groups of low education. Socioeconomically disadvantaged environments and health behaviors related to low educational attainment may be responsible for an altered APOE ɛ4 expression. Higher educated groups seem to be better equipped to reduce their genetic susceptibility for MCI.

**Key messages:**

- Gene-environment interaction seems to be present on the additive scale between education and APOE ɛ4 status on MCI .
- Socioeconomically disadvantaged environments and health behaviors related to low educational attainment may be responsible for an altered APOE ɛ4 expression on MCI.

